# Quantifying the potential renal acid load of edible mushrooms

**DOI:** 10.1038/s41538-024-00259-w

**Published:** 2024-03-12

**Authors:** Maximilian Andreas Storz

**Affiliations:** Department of Internal Medicine II, Centre for Complementary Medicine, Freiburg University Hospital, Faculty of Medicine, University of Freiburg, Freiburg, Germany

**Keywords:** Nutrition, Risk factors

## Abstract

The capability of any food to alter net endogenous acid or base production can be estimated using the potential renal acid load (PRAL) estimation method. The PRAL of edible mushrooms has been rarely examined; thus a quantification study of *n* = 37 edible mushroom species was performed. Results revealed a heterogeneous picture: although the most commonly consumed mushrooms (e.g., *Agaricus bisporus*, *Lentinula edode*s, and *Pleurotus ostreatus* (white)) have alkalizing properties, several acidifying species (*Volvariella volvacea*, *Pleurotus flabellatus*) were identified, which may be attributable to their high phosphorus content.

Diet composition alters humans’ acid–base balance by providing acid or base precursors^1,2^. The majority of plant-based foods generate alkalies, whereas animal-based high-protein foods, such as meats and cheese, have acidifying properties^2,3^. The capability of foods to endogenously alter net acid or net base production after intestinal absorption and metabolism of the respective nutrients is termed potential renal acid load (PRAL) and can be calculated from foods’ nutrient content^1^.

The long-term consumption of high-PRAL diets promotes a subclinical low-grade metabolic acidosis state, which has been associated with systemic inflammation and tissue damage in the human body^2,4,5^. Low-PRAL diets, on the other hand, have been related to improved metabolic parameters and improved anaerobic exercise performance^6–8^. Replacing sulfur-rich animal protein —a major PRAL contributor—with high-quality plant protein may thus be beneficial to human health^5^.

Edible mushrooms are commonly consumed in many countries and are traditionally known as a good protein source^9,10^. They are also low in fat and high in potassium. Mushrooms’ PRAL has been rarely examined, and the original PRAL reference list by Remer et al. only contains a single and not closer specified mushroom type called “common mushrooms”^11^. Due to the high heterogeneity and variability in mushrooms’ nutrient content^9^, a more sophisticated PRAL assessment of edible mushrooms was deemed necessary.

Based on a scientific literature review, we identified a total of *n* = 37 edible mushrooms without missing information on PRAL-relevant nutrients. Table 1 displays their nutrient content and the estimated PRAL scores based on a dry matter basis^9,12–16^.

**Table 1:** Nutrient content and resulting potential renal acid load of selected edible mushrooms based on dry weight

Name	Protein	Calcium	Potassium	Magnesium	Phosphorus	PRAL	Source
*Agaricus bisporus* (Champignon)	26.99	20	3620	117.5	1075	−26.33	^9^
*Agaricus bisporus* (Portobello)	29.78	17.5	3480	117.5	1073	−22.07	^9^
*Agaricus brasiliensis*	33.39	28.75	2000	115	1327	20.10	^9^
*Astraeus hygrometricus* (mature)	14.7	240	1280	160	220	−18.82	^12^
*Astraeus hygrometricus* (young)	14	80	2610	120	570.00	−31.02	^12^
*Auricularia polytricha*	17.44	88.62	294	83.54	623.96	22.13	^13^
*Coprinus cinereus*	17	214	3232	36	1142	−21.01	^14^
*Craterellus aureus*	14.1	14.6	2063.7	105	1901.9	31.02	^15^
*Craterellus aureu*s 2	18.3	30	4520	120	420	−73.92	^12^
*Craterellus odoratus*	15.5	20	2610	50	210	−41.01	^12^
*Flammulina velutipes*	19.01	3.75	2550	152.5	908	−14.65	^9^
*Ganoderma lucidum*	15.04	109.2	742.1	89.1	502.5	6.64	^16^
*Heimiella retispora*	21.1	20	3700	120	600	−48.54	^12^
*Heimiella* sp.	16.3	20	2570	80	330	−36.11	^12^
*Hericium erinaceus*	18.8	11	2912.3	75.81	770.8	−25.28	^16^
*Lactarius glaucescens*	18.6	10	2810	80	530	−32.50	^12^
*Laetiporus sulphureus*	8.62	13.04	433.62	13.85	542.88	14.67	^13^
*Lentinula edodes* (Shitake)	18.87	16.25	2050	155	774	−9.41	^9^
*Phaeogyroporus portentosus*	24.2	30	3330	120	810	−31.61	^12^
*Pleurotus djamor*	22.54	15	2790	175	617	−29.46	^9^
*Pleurotus eryngii*	16.47	10	1860	117.5	787	−5.06	^9^
*Pleurotus flabellatus*	21	120	1537	40	1616	35.21	^14^
*Pleurotus ostreatus* (black)	36.96	15	2690	168.75	1540	14.02	^9^
*Pleurotus ostreatus* (white)	22.54	8.75	3060	148.75	699	−31.33	^9^
*Polyporus dictyopus*	6.6	65.31	239.45	64.47	684.21	21.00	^13^
*Polyporus tenuiculus*	10.89	90.95	428.41	94.48	592.25	14.61	^13^
*Russula alboareolata*	21.2	20	3620	130	660	−44.85	^12^
*Russula lepida*	18.3	10	3530	70	410	−51.94	^12^
*Russula nigricans*	22.6	20	2530	60	340	−31.30	^12^
*Russula virescens*	20	10	2760	80	510	−31.50	^12^
*Russula xerampelina*	22.4	10	2890	60	330	−39.19	^12^
*Sarcodon aspratus*	12	7.6	2790.9	75.2	1780.7	11.10	^15^
*Termitomyces microcarpus*	30.69	37.47	1112.76	39.03	898.17	23.40	^13^
*Termitomyces* sp. 1	28.24	25.93	1179.63	29.11	776.82	16.71	^13^
*Termitomyces* sp. 2	21.26	49.31	1200.28	50.75	925.69	17.50	^13^
*Termitomyces striatus*	21.76	26.39	1450.44	28.47	739.06	6.47	^13^
*Volvariella volvacea*	28	446	1324	57	1699	41.50	^14^

PRAL in mEq/100 g; all minerals are displayed in mg/100 g dry mass; protein in g/100 g dry mass.

*PRAL* potential renal acid load.

The mean PRAL score of all examined mushrooms was −10.83 ± 28.73 mEq/100 g. Approximately 40.5% (*n* = 15/37) of mushrooms displayed acidifying properties (PRAL > 0 mEq/100 g). The highest PRAL values were found for *Volvariella volvacea* (41.50 mEq/100 g), *Pleurotus flabellatus* (35.21 mEq/100 g) and *Craterellus aureus* (31.02 mEq/100 g). Among those with alkalizing properties (PRAL < 0 mEq/100 g) the following mushrooms were noticeable: *Craterellus aureus* (−73.92 mEq/100 g), *Russula lepida* (−51.94 mEq/100 g) and *Heimiella retispora* (−48.54 mEq/100 g).

Mushrooms’ mean protein content was 20.14 ± 6.59 g/100 g. Mushrooms were also characterized by a high potassium (mean: 2264.88 ± 1095.50) and phosphorus content (median: 699 (395.69)) in mg/100 g. As shown in Fig. 1, potassium and phosphorus content were strongly correlated with PRAL (Pearson’s *r*: −0.80 and Spearman’s rho: 0.62, respectively; *p* < 0.001 for both), whereas no significant association was found for protein content.

**Fig. 1:**
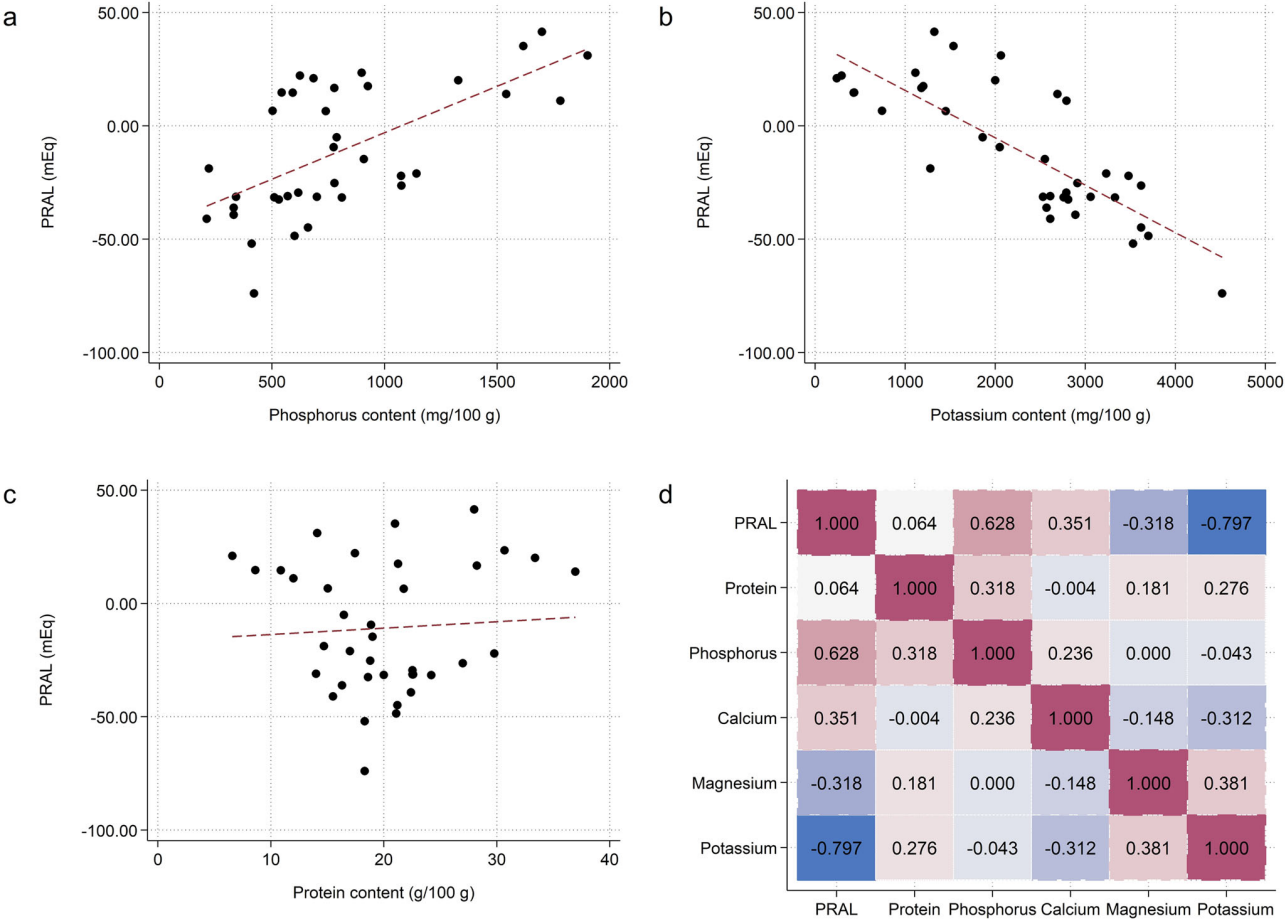
Scatterplots and heatplot showing the relationship between mushrooms’ nutrient content and PRAL. Top row (**a** and **b**) Scatterplots showing correlations between PRAL and phosphorus, and PRAL and potassium, respectively (both in mg/100 g). A strong inverse relationship between the potassium content and PRAL was observed (Pearson’s *r*: −0.80; *p* < 0.001). A strong positive relationship between PRAL and the phosphorus content was observed (Spearman’s rho: 0.62; *p* < 0.001). Bottom row (**c** and **d**) Scatterplot (**c**) showing the non-significant association between PRAL and protein content (in g/100 g). Heatplot (**d**) showing the correlations between the examined minerals (right). Only potassium and phosphorus correlated significantly with the PRAL of edible mushrooms.

The PRAL values of edible mushrooms varied substantially and although the sample’s mean generally indicated alkalizing properties, several acidifying candidates were identified. When specifically glancing at some of the most commonly consumed mushroom types in the Western world (*Agaricus bisporus, Lentinula edodes*, and *Pleurotus ostreatus* (white)), all were characterized by negative PRAL values.

In terms of protein and amino acid composition, mushrooms have been proposed as suitable substitutes for animal-based foods (e.g. meat)^17^. Their PRAL values, however, have been rarely explored and received little attention in the past.

Thus, the herein presented PRAL tables could be helpful for individuals who wish to alkalize their diet and could be of great support for nutritionists who intend to optimize the PRAL of their patients.

While covering an unexplored field, this brief communication does likely not cover all edible mushrooms in the sense of a systematic review. Further to that, nutrient and mineral contents of mushrooms on a dry matter basis were used. This may have led to an overestimation of PRAL when considering fresh mushrooms, which usually have a moisture content of up to 90%^18^. Although drying is one of the most significant preservation methods employed for the storage of mushrooms^19^, they are not exclusively consumed as dried foods. The nutrient content of mushrooms, however, is mostly reported based on a dry matter basis^20^. While such data was employed for comparative purposes here, we clearly acknowledge the potential limitations of this approach. To ensure a transparent comparison, we also provide PRAL values of selected mushrooms based on fresh edible 100 g portions in Table 2 (which is based on data from the U.S. Department of Agriculture^21^).

**Table 2:** Potential renal acid load of selected edible mushrooms based on fresh weight

Name	Water	Protein	Calcium	Potassium	Magnesium	Phosphorus	PRAL
Beech mushroom	89.8	2.18	0.00	376.00	10.50	86.00	−3.92
Crimini mushroom	*91.8*	3.09	4.00	380.00	10.20	100.00	−3.08
Enoki mushroom	88.3	2.42	1.00	402.00	12.80	84.00	−4.49
Maitake mushroom	90.4	2.20	2.49	260.00	11.00	72.00	−2.04
Oyster mushroom	89.2	2.90	2.50	282.00	13.90	86.00	−1.71
Pioppini mushroom	89.6	3.5	2.49	392	16	121	−2.49
Portobella mushroom	91.5	2.75	3	349	9	87	−3.04
Shiitake mushroom	88.6	2.41	1	243	14.1	76	−1.49
King Oyster mushroom	88.1	2.41	2.49	294	13.5	90	−2.05
Lion’s Mane mushroom	88.6	2.5	2.5	443	11.7	94	−4.94
White Button mushroom	91.8	2.89	5	373	10.2	93	−3.31

Based on data from the U.S. Department of Agriculture^21^. PRAL in mEq/100 g; all minerals are displayed in mg/100 g fresh mass; protein in g/100 g fresh mass; water in g/100 g fresh mass. *Note*: the USDA Food Database does not provide binomial names for the abovementioned food items.

*PRAL* potential renal acid load.

Fresh weight-based PRAL values expectedly were much smaller, yet nutrient mushroom content data based on fresh weight is rarely reported in the scientific literature. While potentially less accurate, dry weight-based PRAL data may still be of importance to differentiate between alkalizing and acidifying mushroom species.

Finally, we highlight that some important edible mushrooms, such as *Cantharellus cibarius*, were not included in this analysis because publications that included all PRAL-relevant nutrients for the aforementioned species could not be identified. The same applied to mycelial extracts, for which PRAL-relevant nutrient profiles were only available in a limited number of publications^22^.

Nevertheless, the present results were deemed important. This analysis highlights the heterogeneous PRAL of mushrooms and proposes several mushroom types that allow for two important goals at the same time: substituting animal protein with plant protein while simultaneously optimizing PRAL without diminishing protein intake quantity and quality.

## Methods

### Data gathering

This brief communication is part of a series of short contributions covering the PRAL value of novel, underexplored, or uncommon food groups^5,23^. The nutrient content of selected edible mushrooms was extracted from previous publications, which were identified using PubMed and Google Scholar. The literature search strategy included the following search terms: edible mushrooms; nutrient content; nutritional value; protein; and minerals.

Due to the exploratory character of this brief communication, the literature search was restricted to the aforementioned databases and not designed to reflect a systematic review. Cross-references and reference lists of the identified articles were screened for additional articles to increase the sample size for analysis. Only data from edible mushrooms with a complete nutrient profile required for PRAL estimation (see below) was extracted. Publications that did not contain all PRAL-relevant nutrients were not eligible. Only sources that normalized the nutritional composition of mushrooms according to their dry matter content were included in the primary analysis. Articles that provided the nutritional content in other units (e.g., ppm) were not considered. The search was restricted to English language publications from the last 10 years and the entire review process was conducted by the author in June 2023.

### PRAL estimation

PRAL (in mEq/100 g) was estimated based on the commonly employed formula by Remer et al.^24^; it is shown in Eq. (1) below:

1 $$\begin{array}{ll}{\rm{PRAL}}=&(0.49* {\rm{protein}}({\rm{g}}))+(0.037* {\rm{phosphorus}}\,({\rm{mg}}))-(0.021* {\rm{potassium}}\,({\rm{mg}}))\\&-\,(0.026* {\rm{magnesium}}\,({\rm{mg}}))-(0.013* {\rm{calcium}}\,({\rm{mg}})).\end{array}$$

The PRAL score is a validated method and considers ionic dissociation, intestinal absorption rates for the included nutrients as well as sulfur metabolism^1,5,23^.

### Statistical analyses and procedures

PRAL values were calculated in mEq/100 g dry mass of each edible mushroom. The Shapiro–Wilk test was used to determine whether data was normally distributed or not. The mean ± SD was provided for normally distributed variables, whereas medians and interquartile ranges were provided for non-normally distributed variables. Pearson’s product-moment correlations and Spearman’s rank-order correlations were run to assess the relationship between the content of selected nutrients and PRAL. Nutrient-dependent scatterplots and heat plots were created to graphically display the results. Data was analyzed with STATA 14 statistical software (StataCorp. 2015. Stata Statistical Software: Release 14. College Station, TX: StataCorp LP).

### Reporting summary

Further information on research design is available in the Nature Research Reporting Summary linked to this article.

### Supplementary information

Reporting Summary

## Data Availability

All data generated or analyzed during this study are included in this published article.
