# Supplementary material for: Quantifying the potential renal acid load of edible mushrooms
Source: NPJ Sci Food. 2024 Mar 12;8:17. doi: 10.1038/s41538-024-00259-w (PMC10933430; doi:10.1038/s41538-024-00259-w)
Supplement: Supplementary file 1 — Reporting Summary [file 41538_2024_259_MOESM1_ESM.pdf]

nature portfolio

Corresponding author(s): Dr. Maximilian Andreas Storz

Last updated by author(s): Feb 16, 2024

Reporting Summary

Nature Portfolio wishes to improve the reproducibility of the work that we publish. This form provides structure for consistency and transparency in reporting. For further information on Nature Portfolio policies, see our Editorial Policies and the Editorial Policy Checklist.

Statistics

For all statistical analyses, confirm that the following items are present in the figure legend, table legend, main text, or Methods section.

n/a	Confirmed	
☐	☒	The exact sample size (*n*) for each experimental group/condition, given as a discrete number and unit of measurement
☒	☐	A statement on whether measurements were taken from distinct samples or whether the same sample was measured repeatedly
☒	☐	The statistical test(s) used AND whether they are one- or two-sided<br>*Only common tests should be described solely by name; describe more complex techniques in the Methods section.*
☒	☐	A description of all covariates tested
☐	☒	A description of any assumptions or corrections, such as tests of normality and adjustment for multiple comparisons
☐	☒	A full description of the statistical parameters including central tendency (e.g. means) or other basic estimates (e.g. regression coefficient) AND variation (e.g. standard deviation) or associated estimates of uncertainty (e.g. confidence intervals)
☒	☐	For null hypothesis testing, the test statistic (e.g. F, t, r) with confidence intervals, effect sizes, degrees of freedom and P value noted<br>*Give P values as exact values whenever suitable.*
☒	☐	For Bayesian analysis, information on the choice of priors and Markov chain Monte Carlo settings
☒	☐	For hierarchical and complex designs, identification of the appropriate level for tests and full reporting of outcomes
☒	☐	Estimates of effect sizes (e.g. Cohen's *d*, Pearson's *r*), indicating how they were calculated

Our web collection on statistics for biologists contains articles on many of the points above.

Software and code

Policy information about availability of computer code

Data collection	Not applicable, this is a secondary data analysis of manually extracted data.
Data analysis	Not applicable, this is a secondary data analysis of manually extracted data.

For manuscripts utilizing custom algorithms or software that are central to the research but not yet described in published literature, software must be made available to editors and reviewers. We strongly encourage code deposition in a community repository (e.g. GitHub). See the Nature Portfolio guidelines for submitting code & software for further information.

Data

Policy information about availability of data

All manuscripts must include a data availability statement. This statement should provide the following information, where applicable:

- Accession codes, unique identifiers, or web links for publicly available datasets
- A description of any restrictions on data availability
- For clinical datasets or third party data, please ensure that the statement adheres to our policy

All data generated or analyzed during this study are included in this published article.

Research involving human participants, their data, or biological material

Policy information about studies with human participants or human data. See also policy information about sex, gender (identity/presentation), and sexual orientation and race, ethnicity and racism.

Reporting on sex and gender	Not applicable - this secondary data analysis does not contain human data.
Reporting on race, ethnicity, or other socially relevant groupings	Not applicable - this secondary data analysis does not contain human data.
Population characteristics	Not applicable - this secondary data analysis does not contain human data.
Recruitment	Not applicable - this secondary data analysis does not contain human data.
Ethics oversight	Not applicable - this secondary data analysis does not contain human data.

Note that full information on the approval of the study protocol must also be provided in the manuscript.

Field-specific reporting

Please select the one below that is the best fit for your research. If you are not sure, read the appropriate sections before making your selection.

☐ Life sciences ☐ Behavioural & social sciences ☒ Ecological, evolutionary & environmental sciences

For a reference copy of the document with all sections, see nature.com/documents/nr-reporting-summary-flat.pdf

Ecological, evolutionary & environmental sciences study design

All studies must disclose on these points even when the disclosure is negative.

Study description	The present study is a secondary data analysis based on published scientific literature. We extracted the nutrient content from selected edible mushrooms and calculated the so-called Potential Renal Acid Load (PRAL) score of each mushroom.
Research sample	N = 37 mushrooms were analyzed. The nutrient content of selected edible mushrooms was extracted from previous publications, which were identified using PubMed and Google Scholar. The literature search strategy included the following search terms: edible mushrooms; nutrient content; nutritional value; protein; minerals.
Sampling strategy	All mushrooms with a complete nutritional profile (covering all PRAL-relevant nutrients) were included. To be considered eligible, protein content as well as mineral contents (magnesium, potassium, calcium, phosphorus) had to be reported.
Data collection	Cross-references and reference lists of the identified articles were screened for additional articles to increase the sample size for analysis. Only data from edible mushrooms with a complete nutrient profile required for PRAL estimation (see above) was extracted. Publications that did not contain all PRAL-relevant nutrients were not eligible. Only sources that normalized the nutritional composition of mushrooms according to their dry matter content were included. Articles that provided the nutritional content in other units (e.g., ppm) were not considered. The search was restricted to English language publications from the last 10 years and the entire review process was conducted by the author in June 2023.
Timing and spatial scale	Not applicable.
Data exclusions	Other than specified above, no data was excluded.
Reproducibility	All data necessary to calculate the PRAL of the examined mushrooms can be found in the publication.
Randomization	Not applicable.
Blinding	Not applicable.

Did the study involve field work? ☐ Yes ☒ No

Reporting for specific materials, systems and methods

We require information from authors about some types of materials, experimental systems and methods used in many studies. Here, indicate whether each material, system or method listed is relevant to your study. If you are not sure if a list item applies to your research, read the appropriate section before selecting a response.

Materials & experimental systems

n/a	Involved in the study
☒	☐ Antibodies
☒	☐ Eukaryotic cell lines
☒	☐ Palaeontology and archaeology
☒	☐ Animals and other organisms
☒	☐ Clinical data
☒	☐ Dual use research of concern
☒	☐ Plants

Methods

n/a	Involved in the study
☒	☐ ChIP-seq
☒	☐ Flow cytometry
☒	☐ MRI-based neuroimaging

Plants

Seed stocks	Not applicable.
Novel plant genotypes	Not applicable.
Authentication	Not applicable.
